# Eyelid Closure Behavior of Patients with Idiopathic and Nonorganic Hypersomnia, Narcolepsy-Cataplexy, and Healthy Controls in the Maintenance of Wakefulness Test

**DOI:** 10.2147/NSS.S408318

**Published:** 2023-08-18

**Authors:** Annelies Santschi, David R Schreier, Anneke Hertig-Godeschalk, Samuel E J Knobel, Uli S Herrmann, Jelena Skorucak, Wolfgang J Schmitt, Johannes Mathis

**Affiliations:** 1 Department of Neurology, Inselspital, Bern University Hospital, University of Bern, Bern, Switzerland; 2 University Children’s Hospital Zurich, Zurich, Switzerland; 3 University Hospital of Psychiatry, University of Bern, Bern, Switzerland; 4 Sleep Medicine, Neurozentrum Bern, Bern, Switzerland

**Keywords:** hypersomnia, hypersomnia associated with psychiatric disorders, excessive daytime sleepiness, vigilance test, central disorders of hypersomnolence, microsleep

## Abstract

**Purpose:**

Differential diagnosis of central disorders of hypersomnolence remains challenging, particularly between idiopathic (IH) and nonorganic hypersomnia (NOH). We hypothesized that eyelid closure behavior in the maintenance of wakefulness test (MWT) could be a valuable biomarker.

**Patients and Methods:**

MWT recordings of patients with IH, NOH, narcolepsy-cataplexy (NC), and healthy sleep-deprived controls (H) were retrospectively analyzed (15 individuals per group). For each MWT trial, visual scoring of face videography for partial (50–80%) and full eyelid closure (≥80%) was performed from “lights off” to the first microsleep episode (≥3 s).

**Results:**

In all groups, the frequency and cumulative duration of periods with partial and full eyelid closure gradually increased toward the first microsleep episode. On the group level, significant differences occurred for the latency to the first microsleep episode (IH 21 min (18–33), NOH 23 min (17–35), NC 11 min (7–19), H 10 min (6–25); p = 0.009), the ratio between partial and full eyelid closure duration (IH 2.2 (0.9–3.1), NOH 0.5 (0–1.2), NC 2.8 (1.1–5), H 0.7 (0.4–3.3); p = 0.004), and the difference between full and partial eyelid closure duration in the five minutes prior to the first microsleep episode (∆full – partial eyelid closure duration: IH −16 s (−35 to 28); NOH 46 s (9–82); NC −6 s (−26 to 5); H 10 s (−4 to 18); p = 0.007). IH and NOH significantly differed comparing the ratio between partial and full eyelid closure (p = 0.005) and the difference between ∆full – partial eyelid closure duration in the five minutes prior to the first microsleep episode (p = 0.006).

**Conclusion:**

In the MWT, eyelid closure behavior (∆full – partial) in the period prior to the first microsleep episode could be of value for discriminating NOH from other etiologies of excessive daytime sleepiness, particularly IH.

## Introduction

The term “hypersomnolence” was introduced as an umbrella term in the third version of the international classification of sleep disorders in 2014 (ICSD-3) covering a wide spectrum of subjective symptoms, reaching from excessive daytime sleepiness (EDS) with an irrepressible need to sleep, to prolonged sleep need per 24-h cycle.^1^ EDS is a common complaint with a prevalence of up to 20% in the general population,^2^,^3^ and an even higher prevalence of up to 40% among patients with various mood disorders.^4^ EDS impairs the quality of life by decreasing performance and productivity and is related to cardiovascular diseases and driving accidents.^3^,^5–7^ Hypersomnia, in the strict sense of prolonged sleep need, often results in absence from work due to the great difficulty of waking up in the morning. There are many possible causes for “hypersomnolence”, even when limited to the area of sleep medicine, such as circadian rhythm disorders, sleep-related breathing disorders, insufficient sleep, central disorders of hypersomnolence, as well as the use of medication or drugs.^8^,^9^ Most of these disorders can be diagnosed with reasonable certainty, once patients are examined in a sleep laboratory. However, the differentiation of central disorders of hypersomnolence remains challenging despite extensive diagnostic tools to help objectify the patient’s sleepiness complaint, particularly between narcolepsy without cataplexy, idiopathic hypersomnia, and nonorganic hypersomnia (hypersomnia associated with a psychiatric disorder). According to the International Classification of Sleep Disorders (ICSD-1, −2, and −3),^1^,^10^,^11^ narcolepsy without cataplexy is diagnosed in the presence of a sleep latency <8 minutes in the multiple sleep latency test (MSLT) and ≥ two sleep-onset rapid eye movement sleep periods (SOREMPs) in the MSLT and/or polysomnography (PSG). However, it is widely accepted that this criterion is neither specific nor sensitive and the diagnosis based on these criteria may change over time between, eg, narcolepsy without cataplexy and idiopathic hypersomnia.^12^ The difficulty in the differentiation of idiopathic and nonorganic hypersomnia is the similarity in their clinical presentation and the absence of reliable specific objective clinical features or laboratory biomarkers.^1^,^13^ On the one hand, although mental disorders diagnoses are considered to be an exclusion criterion for diagnosis of idiopathic hypersomnia, psychiatric symptoms not fulfilling a psychiatric diagnosis are often present.^14–16^ On the other hand, hypersomnia and/or EDS may persist as a particularly pertinacious symptom in (partially) remitted depression, and therefore, an MSLT sleep latency below eight minutes (ie, criterion for idiopathic hypersomnia) may not be rare.^16–20^ This pleomorphic clinical picture complicates the reliable determination between psychiatric vs “organic” causes of hypersomnolence, which is of major importance due to their different treatment approaches.^9^,^13^,^17^

Eyelid metrics have been studied during simulated and real driving, the psychomotor vigilance test, and in the MSLT and maintenance of wakefulness test (MWT) and are accepted as indicators of increasing sleepiness and sleep-related performance deficits.^21–28^ Evidence shows an association between increasing sleepiness and sleep-related performance deficits with a change in spontaneous blink frequency,^21^,^25^ an increased blink duration^24^,^28^ extending to prolonged eyelid closures,^21^,^23^,^27^ and increased percentage of time with closed eyelids.^23^,^25^,^26^

While formerly restricted to the fitness-to-drive assessment, in 2005, the MWT (extended by face videography) was incorporated into the clinical routine diagnostic process of the Sleep-Wake-Epilepsy-Centre in Bern for all patients with hypersomnolence.^20^ Furthermore, the scoring of the electroencephalogram (EEG) recorded throughout the MWT did not consist of sleep and wakefulness according to Rechtschaffen and Kales^29^ only but included the scoring of microsleep episodes (MSE) as well.^30^ In the following years, we observed a peculiar eyelid closure behavior during the MWT in some patients with nonorganic hypersomnia; they closed their eyes long before the first MSE or sleep was detected in the EEG. In contrast, patients with severe “organic” sleepiness, like idiopathic hypersomnia or narcolepsy, often kept their eyes partially open while a sleep-like pattern or microsleep episodes were already observed in the EEG.

Therefore, the present study aimed to systematically investigate if eyelid closure behavior in the MWT could contribute to the differentiation between central disorders of hypersomnolence, particularly between “organic” and “nonorganic” causes of hypersomnolence. The MWT was selected because sleepiness, and therefore eyelid closure, is likely to occur and persist since individuals are instructed to stay awake with their eyes open while facing a very monotonous and sleep-promoting environment.^25^,^28^ Furthermore, psychological factors and decreased motivation, reported in nonorganic hypersomnia but not idiopathic hypersomnia,^31–33^ can shorten the sleep latency in the MWT.^34^,^35^ Based on these considerations and our clinical experience, we hypothesized that a difference in eyelid closure behavior would exist between different groups with hypersomnolence. We focused on the comparison between idiopathic and nonorganic hypersomnia, expecting that patients with nonorganic hypersomnia would close their eyes earlier, show more full compared to partial eyelid closure, and cumulatively keep their eyelids closed for longer before the first MSE than those with idiopathic hypersomnia.

The main objectives derived from this aim were to analyze and compare the following within and across our four groups (idiopathic hypersomnia, nonorganic hypersomnia, narcolepsy-cataplexy, and healthy sleep-deprived controls): (I) latencies to first full eyelid closure and the first microsleep episode (MSE), (II) cumulative duration and proportion of partial and full eyelid closure and their change over trial duration up to the first MSE, (III) correlations between eyelid closure and the latency to the first MSE.

## Materials and Methods

### Design and Participants

This retrospective study includes patients who visited the Sleep-Wake-Epilepsy-Centre, Inselspital, Bern University Hospital, from 2005 to 2015. Patients were mostly referred by primary care physicians who already performed standard diagnostics (eg, blood draw) and ruled out common medical disorders explaining patients’ sleep-wake complaints. If standard diagnostics were not performed previously or results were not available, they were performed by our center. Eligibility criteria upon review of medical records were (i) the presence of a detailed medical history, (ii) a completed Epworth sleepiness scale, (iii) availability of a diagnostic MWT recording including face videography (excl. fitness-to-drive MWTs), (iv) absence of sleep-wake-affecting medication at the time of testing (no intake previously or discontinuation for at least 24 hours prior to testing), and (v) diagnosis of either idiopathic hypersomnia, narcolepsy-cataplexy, or nonorganic hypersomnia. Patients diagnosed with more than one diagnosis of interest, ie, idiopathic and nonorganic hypersomnia, were excluded.

In general, the clinical diagnosis was based on ICSD-2 or ICSD-3.^1^,^11^ An exception was made for idiopathic hypersomnia, which was diagnosed exclusively in the presence of prolonged sleep need, ie, idiopathic hypersomnia with long sleep time (>10 hours per night or >11 hours per 24 hours, based on polysomnography or wrist actigraphy and sleep log). This long sleep need was usually associated with great difficulty to hear the alarm clock in the morning and sleep drunkenness after getting up. Reliable discrimination of idiopathic hypersomnia without long sleep time, ie, absence of hypersomnia in the strict sense of extended need for sleep, and narcolepsy without cataplexy is often not possible.^36^,^37^ Consequently, those two very ambiguous diagnoses could have distorted the outcomes of this study, and thus, both were excluded together with those diagnosed as EDS of unknown cause, a group similar to the ICSD-2 diagnosis “Physiological (organic) Hypersomnia, unspecified”.^11^

Patients with nonorganic hypersomnia described the hypersomnolence paralleling their affective symptoms at the beginning of the disease, but in the later course, their affective symptoms were partially remitted in contrast to the persisting hypersomnolence. They complained about a prolonged need for sleep and lack of energy to get out of the bed in the morning, despite hearing the alarm clock. Tiredness, poor work attendance, and social withdrawal were reported by several patients.

Patients with narcolepsy-cataplexy described typical episodes with loss of muscle tone suggesting cataplexy. Objective findings included a reduced hypocretin level <100mcg/L, a sleep latency of <8 minutes in the MSLT, and ≥2 SOREMPs.

Among the eligible patients, we randomly selected 15 for each of the three diagnostic groups (Table 1). An experienced sleep neurologist (JM) verified the clinical diagnoses. An experienced sleep psychiatrist (WJS) additionally verified nonorganic hypersomnia. Hypersomnolence (EDS and/or prolonged sleep need) had to be the main complaint for nonorganic hypersomnia, and a temporal but not necessarily present association to a psychiatric disorder had to be reported. Twenty-eight out of the 30 patients with either idiopathic or nonorganic hypersomnia consulted a psychiatrist and/or completed the Beck Depression Inventory (BDI-II). In the case of one patient with nonorganic hypersomnia, actigraphy and PSG, as well as for two patients with narcolepsy-cataplexy, PSG, respectively, MSLT, data were not available anymore. In the case of one patient with idiopathic hypersomnia and one patient with narcolepsy-cataplexy, PSG data were excluded because they were recorded more than six months away from the MWT recording date.

Patient groups were compared with a sample of 15 healthy sleep-deprived controls, recruited for another study.^38^ They had no history of sleep-wake disorders or medication and underwent the MWT after a full night of sleep deprivation. Actigraphy raw data of healthy controls were corrupted and could not be restored; however, the inactivity index was calculated based on the available (in 13 of 15 healthy controls) sleep-wake diary.

**Table 1:** Demographical, Clinical, and Sleep-Wake Characteristics

	Idiopathic Hypersomnia	Nonorganic Hypersomnia	Narcolepsy-Cataplexy	Healthy Controls
**Individuals, n**	15	15	15	15
**Demographic and clinical data**^a^				
Sex, female:male	10:5	9:6	6:9	7:8
Age, years	25 (21–28)*^†^	39 (28–43)*^†^	32 (26–43)^†^	22 (19–25)^†^
BMI, kg/m^2^	22 (21–26)	25 (22–28)	28 (24–30)	
ESS	15 (14–18)^†^	15 (10–18)^†^	16 (12–17)^†^	6 (5–7)^†^
BDI-II	6 (3–8)* (n = 12)	14 (9–21)* (n = 14)		
**Maintenance of wakefulness test**				
Mean sleep latency, min	26 (18–28)	26 (20–39)	17 (10–33)	16 (10–30)
**Multiple sleep latency test, n**	15	15	13	15
Mean sleep latency, min	5.1 (4.5–7.7)^†^	4.5 (3.8–7.7)^†^	2.5 (1.8–5.4)^†^	
Sleep latency < 8 min, n (%)	12 (80)	11 (73)	12 (93)	
**Actigraphic measures, n**	15	14	15	13
Inactivity Index, %	35 (34–39)	35 (32–37)	34 (30–37)	29 (26–30)^b^
**Polysomnography, n**	14	14	12	
Latency to sleep onset, min	5.5 (4.6–12.8)	7 (3.8–8.9)	3.5 (1.4–5.8)	
Total sleep time, min	451 (408–481)*^†^	416 (355–436)*^†^	388 (341–447)^†^	
Sleep efficiency, %	94 (93–96)	96 (92–98)	88 (86–94)	
Sleep stage N1, %	10 (6–14)	11 (7–18)	13 (11–17)	
Sleep stage N2, %	46 (39–49)	52 (45–54)	43 (36–47)	
Sleep stage N3, %	20 (18–25)	14 (11–23)	18 (12–23)	
REM, %	19 (16–20)	14 (11–17)	14 (12–18)	
PLMS-Index, n/h	4 (1–9)	4 (1–7)	2 (0–16)	
AHI, n/h	2 (1–3)	5 (2–6)	1 (0.7–8)	
**Comorbidities, n (%)**	6 (40)	15 (100)	9 (60)	
Allergy/Asthma	3	2	4	
Headache/Migraine	3	1	1	
Hypertonia, coronary heart disease	0	2	2	
Epilepsy	0	1	0	
Depression	0	11	1	
Sleep disorders	1 PLMS	2 DS, 1 RLS, 1 SAS	3 PLMS, 2 SAS	

**Notes**: Unless specified otherwise, results are reported as median and interquartile range. ^a^Obtained at the time of the maintenance of wakefulness test. ^b^Derived from sleep diary and excluded from group comparison due to only approximate nature. ^†^*P* < 0.05 (group level). *P < 0.05 (in the pairwise comparison between idiopathic and nonorganic hypersomnia).

**Abbreviations**: AHI, apnoea hypopnoea index; BDI-II, Beck Depression Inventory; BMI, body mass index; DS, disturbed sleep; ESS, Epworth sleepiness scale; PLMS, periodic limb movements during sleep; RLS, restless legs syndrome; SAS, sleep apnoea syndrome.

The study was approved by the ethics committee of the canton of Bern, Switzerland (KEK-number 185/06), and conducted following the principles of the Declaration of Helsinki and Swiss Law. Informed consent for healthy controls and general consent for patients (signed when entering the University Hospital) are available. Due to the retrospective nature of the study, no trial registration was necessary.

### Recordings

All PSG, MSLT, and MWT (extended by face videography) recordings were performed on-site (Sleep-Wake-Epilepsy-Centre, Bern University Hospital), according to international standards, and by continuous monitoring of a sleep technician.^39–41^ During the MWT, patients and healthy sleep-deprived controls sat in a semi-darkened room and were instructed to stay awake for as long as possible without performing repetitive movements or mental exercises to maintain wakefulness.^40^ EEG (O1-M2, O2-M1, C3-M2, C4-M1, Cz-M1, F3-M2, F4-M1), EOG (electrooculography, left/right), submental electromyogram (EMG), electrocardiogram (ECG), respiratory flow, and face-centered audio-videography were all simultaneously recorded using RemLogic™ (Embla Systems LLC). The four MWT trials were timed around 10:00, 12:00, 14:00, and 16:00. Trials were terminated after either 40 min of wakefulness, online identification of three consecutive epochs of sleep stage one, or online identification of one epoch of any other sleep stage defined according to the scoring criteria by the American Academy of Sleep Medicine.^42^ Three patients with idiopathic hypersomnia skipped the first MWT trial due to prolonged sleep in the preceding “ad libitum” PSG. For one patient with nonorganic hypersomnia, the eyes were covered by glasses in the last two MWT trials which were excluded accordingly since an evaluation of eyelid closure would have been unreliable.

### Outcome Measures

Eyelid closure was scored and analyzed from “lights off” to the first MSE for each MWT trial. An MSE was defined according to the BERN scoring criteria with a slight deviation of a minimum duration of three instead of one second(s) for an MSE.^30^ MSEs were scored by an experienced sleep scientist (DRS), who was blinded to any clinical information of the patient (particularly diagnosis). In the absence of any MSE, the latency to the first MSE was set to 40 min.

**Figure 1:**
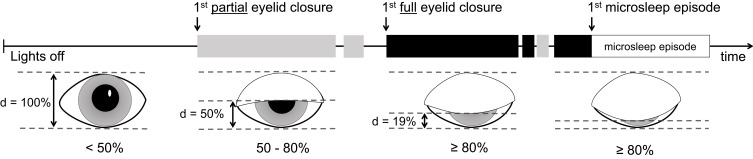
Maintenance of wakefulness tests analysis. Illustration of the different eyelid closure categories and the assessment of eyelid closure behavior during the maintenance of wakefulness test. d = distance, 50–80% = partial eyelid closure (grey shaded block), ≥ 80% = full eyelid closure (black shaded block).

**Figure 2:**
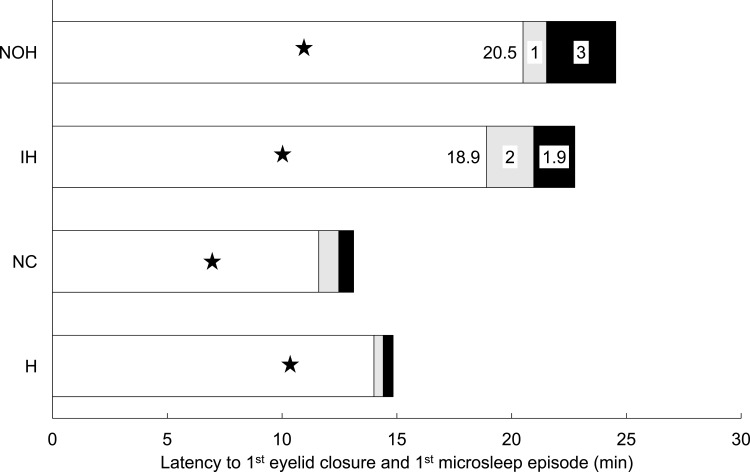
Cumulative duration of eyelid closure. Mean total durations of eyelid closure categories for the three patient groups and healthy controls. The mean latency to the first eye closure is indicated by a star (★) and the mean latency to the first microsleep episode corresponds to the length of the bar. The number within each shaded area of the bar reflects the mean total duration (minutes) of the corresponding eyelid closure category (open = white, partial = grey, full = black).

**Abbreviations**: NOH, nonorganic hypersomnia; IH, idiopathic hypersomnia; NC, narcolepsy-cataplexy.

Eyelid closure was analyzed by continuously scoring periods with partial (50–80%) and full (≥80%) eyelid closure, rated by visual observation in the videography (see Figure 1). Eye blinks, defined as eyelid closure for <1 s, were discarded. Since the total duration of eyelid closure is strongly influenced by trial duration (Figure 2), we calculated eyelid closure duration in relative numbers, ie, in the percentage of the trial duration up to the first microsleep episode. Eyelid closure was visually analyzed by one scorer (AS) and a random subsample of three patients/healthy controls out of each group was analyzed by a second scorer (AHG) for inter-scorer comparison. Both scorers were blinded to the diagnosis and each other’s scoring.

### Statistical Analysis

Stata (StataCorp. 2017, Stata Statistical Software: Release 15.1. College Station, TX: StataCorp LLC), MATLAB (2017b, the Math Works Inc.), Microsoft Excel (Version 15.32, 2017), IBM SPSS (version 26), and GraphPad Prism (V. 8.0.1 (244), November 27, 2018) were used for statistical analysis and graphical illustration.

Not all data were normally distributed, as confirmed by Shapiro–Wilk Test for Normality. The median and interquartile range were reported to summarize the data. Kruskal–Wallis Test with post-hoc Dunn’s test was used for comparative analyses. Spearman’s rho was used for correlation.

With an accuracy of ± 1 s before/after each eyelid closure period scored, inter-scorer reliability was calculated by specificity, sensitivity, and Cohen’s Kappa (poor <0.00, slight 0.00–0.20, fair 0.21–0.40, moderate 0.41–0.60, substantial 0.61–0.80, and almost perfect identification 0.81–1.00).^43–45^

**Figure 3:**
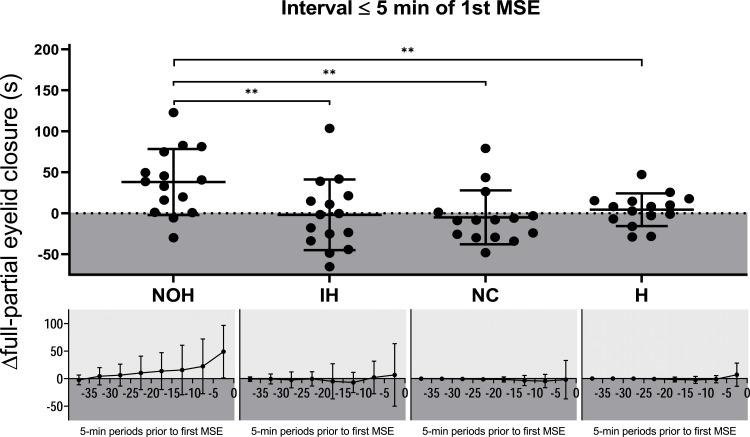
∆ Full – partial eyelid closure. Temporal evolution of the difference between the duration of “full – partial” eyelid closure (y-axis) for each 5-min time interval before the first microsleep episode (MSE, x-axis). The moment of the first MSE is at the right end of the inversed x-axis. A deviation into the positive range means that full eyelid closure dominates, while a downward deviation shows a predominance of partial eyelid closure. The upper figure shows the values of each patient in the last five minutes prior to the first MSE and the significant differences in the pairwise post-hoc comparisons are shown. **P < 0.01.

**Abbreviations**: NOH, nonorganic hypersomnia; IH, idiopathic hypersomnia; NC, narcolepsy-cataplexy; H, healthy controls.

For Table 2 and Figure 3 linear mixed-effect models were used. They were fitted by the restricted maximum likelihood method, and type III sum of squares tests were used to estimate the significance of the effects (procedure MIXED, IBM^®^ SPSS^®^, version 26; documentation extract available in the Supplementary File). Models included intercepts and the only random factor was the patient. The repeated factor was the MWT trial (1–4), whereas fixed factors were diagnosis, MWT trial, and interaction diagnosis*MWT trial. An autoregressive first-order covariance matrix was used for repeated effects, and an identity covariance matrix was used for random effects. We checked the distribution of the residuals, and where necessary, data were log10 transformed (total duration of full and partial eyelid closure, full eyelid closure in relation to trial duration, latency to first full eyelid closure, and ratio partial to full eyelid closure in relation to trial duration). In case the effect of diagnosis was significant, we performed post-hoc comparisons between diagnosis groups, otherwise post hoc comparison was not performed. Since the number of participants per diagnostic group was limited, we refrained from making rigorous corrections for multiple comparisons and the least significant difference (LSD) correction was used. Histograms and q-q plots of residuals are reported in the Supplementary File (Figures S1–S11).

If not indicated otherwise, p < 0.05 (two-tailed) was used as the level of statistical significance.

## Results

### Demographic, Clinical, and Sleep-Wake Data

There were few statistically significant differences between groups regarding demographic, clinical, and sleep-wake data (Table 1). Self-reported sleepiness (Epworth sleepiness scale) differed on a group level due to the, by definition expected, considerably lower scores in healthy controls with similar values across all patient groups. Objective sleepiness, represented by the mean sleep latency in the MSLT, differed on a group level because of the short latency in narcolepsy-cataplexy. The mean sleep latency in the MWT (according to Rechtschaffen and Kales)^29^ and the selected actigraphy variables did not differ across groups. In the PSG, only the total sleep time differed on a group level with the longest value in idiopathic hypersomnia, followed by nonorganic hypersomnia and narcolepsy-cataplexy.

**Table 2:** Eyelid Closure and Microsleep Episodes

	Idiopathic Hypersomnia	Nonorganic Hypersomnia	Narcolepsy-Cataplexy	Healthy Controls	Pairwise Comparison^a^
**Eyelid closure (total trial duration)**					
Latency to first full eyelid closure, min	8 (5–15)	9 (4–13)	4.6 (2–11)	5.5 (3–16)	
Total duration of partial eyelid closure, sec	71 (38–139)	21 (5–73)	44 (16–71)	15 (8–19)	IH-H**, IH-NOH*
Total duration of full eyelid closure, sec	75 (34–172)	115 (33–201)	26 (15–55)	17 (10–38)	IH-H*, NOH-H**, NOH-NC**
Partial eyelid closure in relation to trial duration, %	9 (4–12)	2 (0.4–9)	8 (6–14)	3 (2–6)	IH-H*, NC-H*
Full eyelid closure in relation to trial duration, %	8 (4–13)	11 (5–15)	6 (3–10)	3 (2–7)	
Ratio partial to full eyelid closure in relation to trial duration	2.2 (0.9–3.1)	0.5 (0–1.2)	2.8 (1.1–5)	0.7 (0.4–3.3)	IH-NOH*, NOH-NC**
**Microsleep episodes**					
Number of MWT trials with microsleep episodes	45/57	41/58	54/60	51/60	
Latency to first microsleep episode, min	21 (18–33)	23 (17–35)	11 (7–19)	10 (6–25)	IH-H*, IH-NC*, NOH-H*, NOH-NC**
**Relation of full eyelid closure and microsleep episode**					
Duration from first full eyelid closure to first microsleep episode, min	10 (5–16)	10 (7–15)	5 (1–7)	4 (2–5)	IH-H**, IH-NC**, NOH-H**, NOH-NC*
**Eyelid closure (5-min period prior to first microsleep episode)**					
Partial eyelid closure, s	47 (25–73)	10 (3–35)	27 (14–46)	9 (6–16)	IH-NOH**, IH-H**, NC-H*
Full eyelid closure, s	37 (26–59)	62 (31–104)	20 (13–43)	16 (8–30)	IH-H*, NOH-H**, NOH-NC**
∆ Full – partial eyelid closure, s	−16 (−35–28)	46 (9–82)	−6 (−26–5)	10 (−4–18)	IH-NOH**, NOH-H**, NOH-NC**
**Correlation of first microsleep episode latency to eyelid closure**					
Latency to first full eyelid closure	0.5**	0.7**	0.8**	0.9**	
Partial eyelid closure in relation to trial duration	−0.3*	−0.2	−0.4**	−0.3*	
Full eyelid closure in relation to trial duration	−0.3**	−0.3*	−0.4**	−0.5**	

**Notes**: Unless specified otherwise, results are expressed as median and interquartile range. ^a^Provided that the effect of diagnosis in mixed model analysis revealed *P* < 0.05, pairwise post-hoc comparisons were performed (least significant difference correction for multiple comparisons). *P < 0.05. **P < 0.01.

**Abbreviations**: H, healthy controls; IH, idiopathic hypersomnia; MWT, maintenance of wakefulness test; NC, narcolepsy-cataplexy; NOH, nonorganic hypersomnia.

In the pairwise comparison of idiopathic and nonorganic hypersomnia, patients with idiopathic hypersomnia were younger, BDI-II scores were lower, and the total sleep time in the PSG was longer compared to patients with nonorganic hypersomnia. Notably, the mean sleep latency in the MSLT and the MWT did not differ in the pairwise comparison, underlining that many patients with nonorganic hypersomnia are objectively sleepy.

### Inter-Scorer Reliability

For the 12 randomly selected patients/controls analyzed by two investigators, inter-scorer reliability performance was moderate to substantial, with particularly high specificity and rather high sensitivity for full eyelid closure (Table 3).

**Table 3:** Performance Calculations for Inter-Scorer Reliability

	Sensitivity	Specificity	Cohen’s Kappa
**Partial eyelid closure (50–80%)**	0.55	0.97	0.49
**Full eyelid closure (> 80%)**	0.89	0.94	0.57
**Any eyelid closure (50–100%)**	0.84	0.94	0.71

### Eyelid Closure and Microsleep Episodes

In a majority of patients, ≥1 MSEs occurred in all MWT trials (idiopathic hypersomnia = 9, nonorganic hypersomnia = 8, narcolepsy-cataplexy = 11, healthy controls = 11; Table 2). In a lower number of patients, ≥1 MSE occurred in one to three MWT trials (idiopathic hypersomnia = 6, nonorganic hypersomnia = 3, narcolepsy-cataplexy = 4, healthy controls = 3). Only in four patients with nonorganic hypersomnia and one healthy control, no MSE occurred.

The latency to the first full eyelid closure did not significantly differ across groups. In contrast, the latency to the first MSE was longer in idiopathic and nonorganic hypersomnia compared to narcolepsy-cataplexy and healthy sleep-deprived controls, while it did not differ between idiopathic and nonorganic hypersomnia. The same applies to the duration between the first full eyelid closure and the first microsleep episode (Figure 2, Table 2).

The total duration of partial and full eyelid closure significantly differed across groups (Figure 2, Table 2), but when calculated in relation to trial duration, only the difference in partial eyelid closure (in % of trial duration) remained significant across groups. The ratio of partial to full eyelid closure (in % of trial duration) significantly differed across groups and between idiopathic and nonorganic hypersomnia (Table 2).

In idiopathic hypersomnia, there was a moderate and for the other three groups there was a strong positive correlation between the latency to the first full eyelid closure and the first MSE (Table 2). Furthermore, weak to moderate negative correlations were found for the latency to the first MSE and the cumulative duration of full eyelid closure (in % of trial duration), and – except for nonorganic hypersomnia – the latency to the first MSE and the cumulative duration of partial eyelid closure (in % of trial duration).

**Figure 4:**
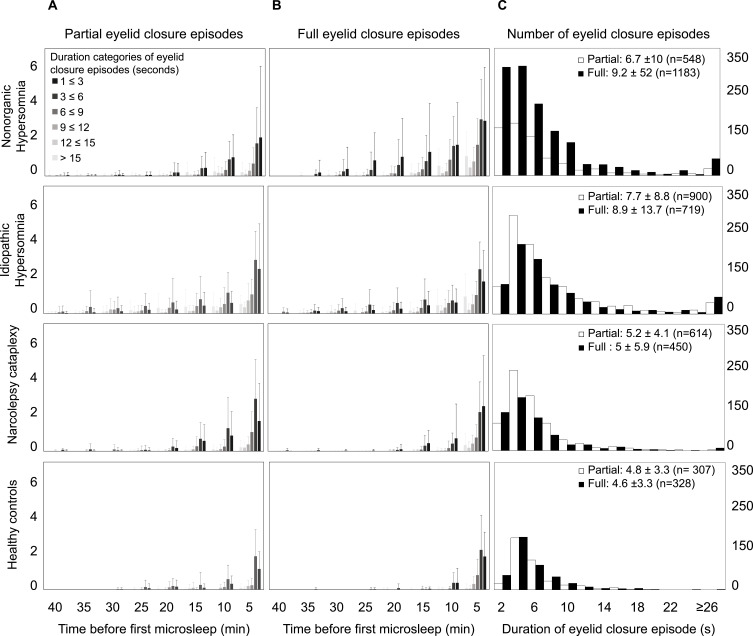
Distribution of eyelid closure episodes. The average frequency of partial (**A**) and full (**B**) eyelid closure episodes per patient and MWT trial for each time interval before the first microsleep episode, divided according to duration categories of eyelid closure episodes (seconds) are shown. In the last column (**C**), the histogram of the duration of individual eyelid closure episodes over all four MWT trials is shown. On the x-axis, the categories of eyelid closure durations in seconds are shown while on the y-axis, the number of eyelid closures in each category can be found (y-axis on left and right). 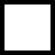 Partial eyelid closure (50–80%); ■ full eyelid closure (>80%).

In patients with narcolepsy-cataplexy and healthy controls, around 90% of partial or full eyelid closure periods lasted <10s, and a duration of >18s was exceeded in only 1–2% of eyelid closure periods (Figure 4C). In patients with nonorganic hypersomnia and idiopathic hypersomnia, 80–85% of partial or full eyelid closure periods lasted <10s, and a duration of >26s was exceeded in ~5% of eyelid closure periods. The most frequent duration of a partial or full eyelid closure episode was 2–4s in all groups.

In all four groups, the frequency of partial and full eyelid closure episodes of any duration, as well as the average duration of partial and full eyelid closure per time period (s/min) increased exponentially towards the first MSE (Figures 4A and B, S12). More than 50% of partial and full eyelid closure episodes occurred in the five minutes preceding the first MSE in all four groups. Within this 5-minute period prior to the first MSE, the cumulative duration of partial eyelid closure was significantly longer in idiopathic compared to nonorganic hypersomnia (Table 2). In contrast, the cumulative duration of full eyelid closure was the longest in nonorganic hypersomnia but did not differ from that of idiopathic hypersomnia. However, the difference between the cumulative duration of “full – partial” eyelid closure in nonorganic hypersomnia was significantly higher compared to all other groups in the 5-min period prior to the first MSE (Figure 3, Table 2). In line with that, full eyelid closure periods occurred more frequently than partial eyelid closure periods in patients with nonorganic hypersomnia, while the occurrence was more equally balanced in the other groups (Figure 4).

## Discussion

To our knowledge, this is the first study to demonstrate that the analysis of eyelid closure in the MWT has the potential to contribute to the differentiation of central disorders of hypersomnolence, in particular between idiopathic and nonorganic hypersomnia, not distinguished by many common biomarkers. In all four investigated groups (idiopathic hypersomnia, nonorganic hypersomnia, narcolepsy-cataplexy, and healthy sleep-deprived controls), more than 50% of partial and full eyelid closure episodes occurred in the 5-min period prior to the first MSE. The difference between the cumulative duration of “full – partial” eyelid closure in this time period was significantly higher in nonorganic hypersomnia compared to all other groups, which might contribute to differentiating nonorganic hypersomnia from other central disorders of hypersomnolence.

According to the current version of the International Classification of Sleep Disorders (ICSD-3), only two neurophysiological variables are relevant in the diagnostic process of central disorders of hypersomnolence: sleep latency and SOREMPs.^1^ Both variables are mainly determined in the MSLT (in combination with the PSG), which aims to primarily measure the level of sleepiness or non-REM- and REM-sleep pressure. In contrast, the MWT is neither required nor recommended as an additional diagnostic tool for any diagnoses. Nevertheless, the MWT is frequently used in clinical sleep medicine due to its ability to assess the capacity to maintain wakefulness, which the MSLT is not designed for. The main purposes are treatment control and optimization but also evaluation of fitness to drive in patients with EDS.^46–49^

A decreased motivation to maintain wakefulness, when instructed to do so, may shorten the sleep latency in the MWT.^50^ To which extent personality influences motivation (independent of the diagnosis) and to which extent both personality and motivation are affected by disorders has still to be determined. It could be speculated that motivation in patients with mood disorders, the most prevalent mental disorder in nonorganic hypersomnia, is lower, and therefore the sleep latency in the MWT is shorter and more similar to the sleep latency in patients with idiopathic hypersomnia.^14^,^51–53^ In this study, patients with idiopathic and nonorganic hypersomnia showed similar sleep latencies in the MWT and the MSLT. The results suggest that full in relation to partial eyelid closure in the 5-min period prior to the first MSE is much more prominent in patients with nonorganic hypersomnia compared to, eg, idiopathic hypersomnia. Therefore, it might be that motivation in patients with nonorganic hypersomnia was diminished, resulting in a reduced capacity to resist eyelid closure, most prominently close to sleep onset. However, the interaction between “organic” and “nonorganic” hypersomnia, motivation, and personality traits is certainly complex and overall poorly investigated. Based on the findings of this study and a previous study investigating the diagnostic value of the Bern vigilance battery (Mathis & Andres et al),^20^ we believe that the diagnostic potential of the MWT should be further explored.

Besides pioneering the detailed analysis of the MWT in the context of the diagnostic process, this study also assessed the simultaneous inclusion of eyelid closure and the first MSE. Despite our more refined classification of the borderland between wakefulness and sleep, including MSEs as short as one second,^30^ we decided to include only MSEs of ≥3 s in this study. The two main reasons were that (i) we aimed to increase specificity for “sleep onset” and (ii) we considered practicability for the clinical routine assessment. Shorter MSEs, ie, <3 s, are more difficult and time-consuming to score. In this study, the first full eyelid closure was an early indicator of sleepiness and the moderate to strong positive correlation with the latency to the first MSE (Table 2) points to a certain association between those two events. Additional analyses of eyelid closure behavior in this time period, between the first full eyelid closure and the first MSE in the MWT, might reveal additional biomarkers for the differentiation of idiopathic and nonorganic hypersomnia. This is of potential clinical relevance since a more accurate diagnosis might inform (etiology-based) treatment.

The findings of this study could contribute to the question of how to assess sleepiness in general, outside of the MWT. Several studies have already investigated the utility of eyelid metrics as indicators of sleepiness and reported similar results to our study. Alvaro et al^21^ reported a progressive increase towards longer and more frequent eyelid closures after 17 h of sustained wakefulness in professional drivers. Furthermore, the cumulative duration of eyelid closure per hour showed a positive correlation with subjective sleepiness and performance impairments in simulated driving (variation in lane position, braking reaction time, crashes, variation in speed) and the psychomotor vigilance test (lapses, reaction time). Similarly, Jackson et al^23^ reported an increase in the proportion of time with eyelid closure after sleep deprivation and a positive correlation of eyelid closure measures with subjective sleepiness, crashes in a simulated driving scenario, and lapses in the psychomotor vigilance test. This illustration of the relationship between eyelid closure and performance highlights the fact that sleepiness is a multi-faceted construct, which can and should be assessed from different perspectives. However, the use of an in-laboratory EEG recording setup is not practical for daily life, eg, a real-driving condition. This is the major reason why the identification of MSEs remains a challenge in real life. In contrast, wireless devices that serve the purpose of identifying eyelid closure exist and the number of those installed in motor vehicles could increase soon since the European Parliament decided that devices detecting drowsiness and distraction have to be installed in any new motor vehicle since May 2022.^54^ The fact that these devices can be used in a laboratory setting and real life allows a much faster and reliable transfer and validation of in-laboratory to real-life situations but could work the other way around as well. The need for such in-vehicle devices accelerates the development and optimization of methods to detect and evaluate eyelid-related metrics. Such in-vehicle or similar devices could then again be used for in-laboratory research and eventually a clinical setting. Reliable and automated detection of eyelid closure would enable to replicate previous studies, such as this one, and potentially represent a fast and objective tool to further support physicians in their clinical work.

The primary limitation of this study is the visual analysis of eyelid closure, which is inevitably subjective to a certain degree, apart from being very time-consuming. Even though inter-scorer reliability was moderate to substantial, it is possible that the overall impression of a patient’s behavior during an MWT trial (yawing, gestures, head movements) subconsciously influenced the scorer’s judgment of the eyelid closure behavior, especially in the differentiation between partial and full eyelid closure. This limitation could be removed by a reliable and automated analysis of eyelid closure, which was not available for retrospective video analysis. In addition, such an approach would allow the inclusion of a bigger set of eyelid metrics, including eyelid closure speed, blink frequency, and blink duration.

Furthermore, the intra-group and intra-individual trial variances were high, suggesting that a wide range of eyelid closure behavior exists. Based on our clinical experience, some patients with disease-typical nonorganic hypersomnia show a rather early full eyelid closure compared to patients with organic causes of EDS, followed by a rather long time period until sleep onset. Large inter-individual differences were already reported in studies investigating the association between sleepiness and eyelid metrics.^24^,^55^ In the present study, the group sizes were too small to perform a subgroup analysis. This leads to another limitation of this study: the small sample size could have been too small to generate sufficient statistical power to better differentiate the selected patient groups, in particular idiopathic and nonorganic hypersomnia. However, the availability of patient data is often a problem in rare disorders and the fact that only a few sleep-wake centers perform the MWT as part of the clinical diagnostic routine in treatment naïve patients, frequently without face videography, limits the data availability to an even greater extent. Furthermore, instead of being two completely separated entities, idiopathic and nonorganic hypersomnia might represent a continuum. This could explain why some measures may overlap or some patients can even be diagnosed with both disorders simultaneously. In this study, patients with unclear diagnosis or co-existing diagnosis of both idiopathic and nonorganic hypersomnia were excluded and thus the overlap between the two disorders reduced. This explains why none of the patients with idiopathic hypersomnia in this study were additionally diagnosed with depression and to some extent why BDI-II was significantly lower in idiopathic compared to nonorganic hypersomnia. Despite our efforts to exclude ambiguous cases, when verifying our data at the end of our study, we noted that the diagnosis of two patients with idiopathic hypersomnia had changed since the inclusion in our study. Both patients were re-diagnosed, one with nonorganic hypersomnia and the other one with narcolepsy-cataplexy. Despite this discovery, we did not change the initial diagnosis of these two patients because diagnostic uncertainty is a reality in clinical sleep medicine and we did not want our data to be influenced by subsequent tests and findings. In our clinical experience, rare cases exist where patients with symptoms fulfilling all criteria for idiopathic hypersomnia seem to be “cured”. For example, one patient at our center who was diagnosed with idiopathic hypersomnia reported that his hypersomnolence suddenly disappeared, after many years of complaining about it. The only change in the medical history was that he had found a new girlfriend, rather suggesting a nonorganic instead of idiopathic hypersomnia in retrospect. Many patients with nonorganic hypersomnia describe themselves as “long sleepers”, long before sleepiness became a health problem. This could indicate that nonorganic hypersomnia may more often evolve in long sleepers or patients with pre-existing idiopathic hypersomnia when experiencing psychosocial stress, while normal or short sleepers may rather develop insomnia in a similar situation.

Finally, our study is limited by the fact that patients with narcolepsy without cataplexy (type 2) and patients with idiopathic hypersomnia without long sleep time were excluded. Since a tool to differentiate nonorganic hypersomnia from these groups would be of particular interest, it is debatable if the decision to exclude these groups was appropriate. However, we preferred to include patients with the most reliable diagnosis for this study, and even with this approach, subsequent reclassifications were observed. Future studies with larger patient populations, a more objective assessment of eyelid closure behavior, and objective measures of EEG-derived sleepiness are needed to include and investigate all groups within the broad spectrum of central disorders of hypersomnolence.

## Conclusions

The analysis of eyelid closure behavior in the MWT could support clinicians in the differentiation of nonorganic hypersomnia from other central disorders of hypersomnolence, eg, idiopathic hypersomnia. Most prominently, the difference between the cumulative duration of full and partial (full – partial) eyelid closure was significantly higher in nonorganic hypersomnia compared to all other groups in the 5-min period prior to the first MSE. Furthermore, eyelid closure and other eyelid metrics seem to be interesting but underestimated variables for the determination of sleepiness and differential diagnosis as part of a vigilance battery. However, future larger studies using a reliable and automated approach to determine eyelid closure are needed to replicate our findings.

## Abbreviations

BDI, Beck Depression Inventory; BERN, Bern continuous and high-resolution wake-sleep criteria for visual MSE scoring; ECG, electrocardiogram; EDS, excessive daytime sleepiness; EEG, electroencephalogram; EMG, electromyogram; EOG, electrooculography; ICSD, International Classification of Sleep Disorders; MSE, microsleep episode; MSLT, multiple sleep latency test; MWT, maintenance of wakefulness test; PSG, polysomnography; SOREMPs, sleep-onset rapid eye movement sleep periods.

## Data Sharing Statement

Due to the privacy protection of patients, the videos that were analyzed cannot be made publicly available. Further data from this study are available upon reasonable request.
